# Clinical and dosimetric predictors of late rectal bleeding of prostate cancer after TomoTherapy intensity modulated radiation therapy

**DOI:** 10.1002/jmrs.217

**Published:** 2017-02-01

**Authors:** Ryoko Katahira‐Suzuki, Motoko Omura, Shoko Takano, Kengo Matsui, Hideyuki Hongo, Wataru Yamakabe, Hironori Nagata, Harumitsu Hashimoto, Ichiro Miura, Tomio Inoue

**Affiliations:** ^1^ Radiation Oncology Shonan Kamakura General Hospital Kamakura Kanagawa Japan; ^2^ Radiology Yokohama City University Medical Center Yokohama Kanagawa Japan; ^3^ Radiology Yokohama City University Yokohama Kanagawa Japan; ^4^ Radiation Oncology Shonan Fujisawa Tokusyukai Hospital Fujisawa Kanagawa Japan; ^5^ Urology Shonan Kamakura General Hospital Kamakura Kanagawa Japan

**Keywords:** Adverse effects, cancer of prostate, gastrointestinal haemorrhage, intensity modulated radiotherapy, radiotherapy dosage

## Abstract

**Introduction:**

Rectal bleeding after radiotherapy impacts the quality of life of long‐term surviving prostate cancer patients. We sought to identify factors associated with late rectal bleeding following intensity modulated radiation therapy (IMRT) using TomoTherapy for prostate cancer.

**Methods:**

We retrospectively analysed 82 patients with localised prostate cancer treated with TomoTherapy. Most patients (95.1%) received neoadjuvant and concurrent hormone therapy. Forty‐two patients (51.2%) graded as high risk using D'Amico's classification underwent radiotherapy involving the pelvic nodal area. Late bleeding complications were quantified using the Common Terminology Criteria for Adverse Events v4.0. Multiple clinical and dosimetric factors were considered with reference to rectal bleeding.

**Results:**

The median follow‐up period was 538 (range, 128–904) days. Grades 1, 2 and 3 rectal bleeding were observed in 14 (17.1%), four (4.9%) and one (1.2%) patient respectively. In multivariate analysis, the following factors were significantly associated with Grade ≥1 late rectal bleeding: volume, mean dose (*P* = 0.012) and rectal V30 (*P* = 0.025), V40 (*P* = 0.011), V50 (*P* = 0.017) and V60 (*P* = 0.036). When exclusively considering Grade 2–3 rectal bleeding, significant associations were observed with the use of anticoagulants or antiaggregates (*P* = 0.007), rectal V30 (*P* = 0.021) and V40 (*P* = 0.041) in univariate analysis.

**Conclusions:**

Our results suggested that the intermediate rectal dose‐volume (V30–V60) was a significant predictor for mild to severe late rectal bleeding (Grade ≥1). Rectal dose‐volumes >V70, which represented the volume of the highest doses, were not predictive in this study.

## Introduction

In the late 1980s, the advent of three‐dimensional conformal radiotherapy (3D‐CRT) enabled the prescription of more conformal and higher dose distribution to targets close to critical organs. The prostate is one of the organs that is surrounded by numerous critical organs, such as the rectum and bladder, and this improvement in radiotherapy technique is certainly a merit of this modality. Several studies have suggested the benefit of dose escalation for the treatment of prostate cancer.^1^, ^2^, ^3^

Unfortunately, dose escalation using 3D‐CRT may be achieved at the expense of increased rectal toxicity. With 3D‐CRT, 15–35% of patients who are treated with a total radiation dose >70 Gy to the prostate have been reported to develop ≥Grade 2 rectal toxicity.^4^ The emergence of intensity modulated radiation therapy (IMRT) has enabled the delivery of a more conformal dose with rapid dose fall‐off around the critical organs; this has resulted in decreased complication rates. The rate of late Grades 2 and 3 rectal bleeding has been found to be reduced from 10% using 3D‐CRT to 2% using IMRT.^5^

However, even in the IMRT era, a small number of patients still experience rectal toxicity after treatment. Because of the impact of rectal bleeding on the quality of life of long‐term surviving patients, it has been continuously investigated and reported.^6^ Several investigators have demonstrated serial‐like behaviour regarding rectal bleeding, especially when considering severe bleeding; this means that relatively small fractions of rectum irradiated with ‘high’ doses are the main cause of bleeding.^7^, ^8^, ^9^, ^10^ In contrast, the impact of intermediate doses on rectal bleeding remains unknown.

The TomoTherapy (TomoTherapy^®^, Accuray, Madison, WI, USA) system has been developed exclusively for IMRT and its use has spread in the last 10 years. It provides rotational delivery of radiation along helical using thousands of beamlets, which can result in better dose conformity to the target. TomoTherapy also incorporates a megavoltage CT that allows for daily CT localisation; this reduces daily set up error. However, the appropriate dose constraints required for a reduction in the rate of complications associated with rectal bleeding still remain to be investigated in this system.

Understanding the factors associated with late rectal bleeding are important in facilitating the development of more tailored treatments on an individual basis. In this study, we analysed clinical and dosimetric data to determine the factors that were most predictive of late rectal bleeding in prostate cancer patients treated with TomoTherapy IMRT.

## Methods

### Patients

A single institutional registry consisting of 88 consecutive patients with clinically localised prostate cancer, who underwent radiotherapy at Shonan Kamakura General Hospital from April 2011 to December 2012, was included in this retrospective study. Six patients were excluded because they had received an additional radiation boost to the tumour site within the prostate. Most patients (95.1%) had undergone neoadjuvant hormone therapy for at least 6 months before the start of radiotherapy, and continued to receive it during radiotherapy. Rectal bleeding complications were recorded at 3–6‐month intervals during the regular follow‐up visits. In this study, we defined late rectal bleeding if it occurred more than 3 months after the end of radiotherapy. Late rectal bleeding was quantified using the Common Terminology Criteria for Adverse Events v4.0. Grades 1–3 were defined as follows: Grade 1, mild symptoms (intervention not indicated); Grade 2, moderate symptoms (medical intervention or minor cauterisation indicated); and Grade 3, transfusion (radiologic, endoscopic or elective operative intervention indicated). Patient clinical characteristics are summarised in Table 1. All patients had given their informed consent before treatment, and for the use of their records for retrospective evaluation of data. Our institutional ethics committee approved this retrospective study.

**Table 1:** Baseline and treatment characteristics of patients (*n* = 82)

Characteristics	Patients, *n*	%
Median age	74 (57–85) years	
Age (years)		
<64	8	9.8
≥64	74	90.2
T stage		
≤T2b	35	42.7
≥T2c	47	57.3
Gleason's score		
≤6	10	12.2
7	40	48.8
≥8	31	37.8
Undetermined	1	1.2
D'Amico classification		
Low	10	12.2
Intermediate	14	17.1
High	58	70.7
Hormonal therapy		
Yes	78	95.1
No	4	4.9
Hypertension		
Yes	33	40.2
No	49	59.8
Diabetes		
Yes	12	14.6
No	70	85.4
Use of anticoagulants or antiaggregants		
Yes	18	22.0
No	64	78.0
Pelvic nodal irradiation		
Yes	42	51.2
No	40	48.8

### Planning optimisation and treatment delivery

In an attempt to minimise internal organ motion during CT simulation and daily treatment, patients were asked to ensure that their rectum was empty; after voiding completely, they drank 300–500 ml of water 30 min in advance of scanning. Planning CT was achieved using an 8‐section CT scanner (GE LightSpeed) with a slice thickness of 2.5 mm. Pelvic MRI was carried out at the same time as the planning CT; the images were fused with each other using Pinnacle^3^ in which the contouring volume of the target and organ at risk were completed.

The prostate and proximal seminal vesicle were considered as the clinical target volume (CTV). The planning target volume (PTV) was generated based on the CTV and a margin of 1 cm in all directions, with the exception of the posterior and inferior margins which were 0.6 cm in all directions. The pelvic lymph node region was contoured with reference to the Radiation Therapy Oncology Group (RTOG) contouring atlas^11^ and the margin was set at 0.3 cm in all directions.

The rectum was defined as a solid organ in transverse planes where the PTV existed, that is, from the highest to the lowest of these planes. We used PTV confined to prostate and proximal seminal vesicle when we defined the rectum. This definition of rectum was different from RTOG guidelines. Planning optimisation and treatment delivery was conducted using TomoTherapy and the Hi‐Art planning system version 4.2.2. A field dimension of 2.5 cm and a modulation factor of 2.0–3.5 were used for most patients. Pitches of 0.287 and 0.430 were used for the PTV and pelvic lymph node region respectively. The standard prescribed dose used in this study for all patients was 76 Gy to the PTV. The dose to the pelvic lymph node region was 46 Gy for patients who were graded as having high‐risk disease using the D'Amico classification, and were aged <80 years at the start of their radiotherapy.

Forty patients (48.8%) were treated with a total dose of 76 Gy in 2 Gy fractions to only the PTV. The other 42 patients (51.2%) were treated first with a total dose of 50 Gy in 2 Gy fractions to the PTV, with concurrent pelvic lymph node irradiation at a total dose of 46 Gy in 1.84 Gy fractions. The PTV then received an additional cone‐down dose of 26 Gy in 2 Gy fractions, bringing the total to 76 Gy, with the exception of two patients who received total doses of 70 and 74 Gy.

All of the doses were prescribed to 50% of the PTV. Each planning goal was to deliver ˃99% and ˃95% of the prescribed dose to ˃87% and ˃95% of the PTV and pelvic lymph node region respectively. The following rectal dose constraints were aimed at accomplishing the following doses to the rectal volume: V76 ≤ 1%; V75 ≤ 15%; V70 ≤ 25%; V60 ≤ 35%; and V40 ≤ 65% (V40–V76: the percentage of fractions of the rectum receiving ≥40–76 Gy). Although we prioritised the PTV and pelvic lymph node dose coverage, we complied with the rectum dose constraints as strictly as possible; we especially attached importance to rectal V75 or V76. The dose constraints of the other organs at risk were as follows: bladder (V75 ≤ 25%, V70 ≤ 35%, V55 ≤ 45% and V40 ≤ 60–65%), small intestine (V60 < 0.5 cc), large intestine (V65 < 0.5 cc) and femoral heads (maximum dose <50 Gy). We also aimed not to exceed 110% of the prescribed dose as maximum dose to the body.

Megavoltage CT (MVCT) was undertaken on a daily basis. If daily MVCT revealed inconsistencies, such as decreased bladder filling, re‐planning using updated CT was performed. If there was unacceptable rectum distention, we used rectal catheter or enema to reduce the distention to achieve a rectal volume similar to planning CT. Twenty patients (24.4%) underwent re‐planning. The dose distributions for each plan were combined on the latest planning CT and the total dose‐volume histogram was evaluated using MIM maestro v6.0.

### Statistical analysis

The following clinical and dosimetric data were considered for each patient: age, T stage, D'Amico classification, hormonal therapy, use of anticoagulants or antiaggregates, pelvic node irradiation, coexistence of hypertension or diabetes, volume of prostate/PTV/rectum, maximal/mean dose, and rectal V30, V40, V50, V60, V70, V75 and V76. The relationship of each factor with Grade ≥1 late rectal bleeding was determined by means of Kaplan–Meier curves. Comparisons were made using the log‐rank test. Potential prognostic factors (which showed *P* < 0.10) were additionally evaluated in multivariate analysis using the Cox proportional hazards model. Cut‐off values for each dosimetric variable for correlation with the risk for Grade ≥1 late rectal bleeding were determined using ROC curves and the Youden Index (sensitivity + and specificity − 1).

Because there was a small number of patients with Grade 2–3 late rectal bleeding, a different method of analysis was applied. The correlations of each factor with the risk of only Grade 2–3 late rectal bleeding were assessed using univariate analysis by means of the independent samples *t* test for continuous variables and Fisher's exact test for categorical variables. In all analyses, differences with *P* ≤ 0.05 were considered statistically significant. Data analysis was performed using SPSS Statistics version 22, (IBM, Chicago, IL, USA).

## Results

Median follow‐up time was 538 (range, 128–904) days. Nineteen patients (23.2%) had Grade ≥1 late rectal bleeding with a median timing for occurrence of 339 days (range, 103–779 days). Grade 1, 2 and 3 rectal bleeding were observed in 14 (17.1%), four (4.9%) and one (1.2%) patients respectively.

### Patient dosimetric data

Patient dosimetric data are summarised in Table 2. The mean values of all the relevant dose‐volume rectal values were within our rectum dose constraints, with the exception of the V76, which was higher than our predetermined constraint.

**Table 2:** Patient dosimetric data (*n* = 82)

Characteristics	Mean ± SD	Dose constraints
Prostate volume (cc)	25.56 ± 12.56	–
PTV volume (cc)	103.51 ± 27.01	–
Rectal volume (cc)	30.14 ± 7.95	–
Rectal maximal dose (Gy)	77.25 ± 0.78	–
Rectal mean dose (Gy)	47.04 ± 5.67	–
Rectal V30 (%)	78.79 ± 13.88	–
Rectal V40 (%)	59.56 ± 13.92	≤65%
Rectal V50 (%)	43.89 ± 11.96	–
Rectal V60 (%)	29.65 ± 9.09	≤35%
Rectal V70 (%)	14.00 ± 5.54	≤25%
Rectal V75 (%)	3.88 ± 2.87	≤15%
Rectal V76 (%)	1.69 ± 1.80	≤1%

PTV, planning target volume; V30–V76, volume receiving ≥30–76 Gy.

### Patients with Grade ≥1 late rectal bleeding

**Figure 1:**
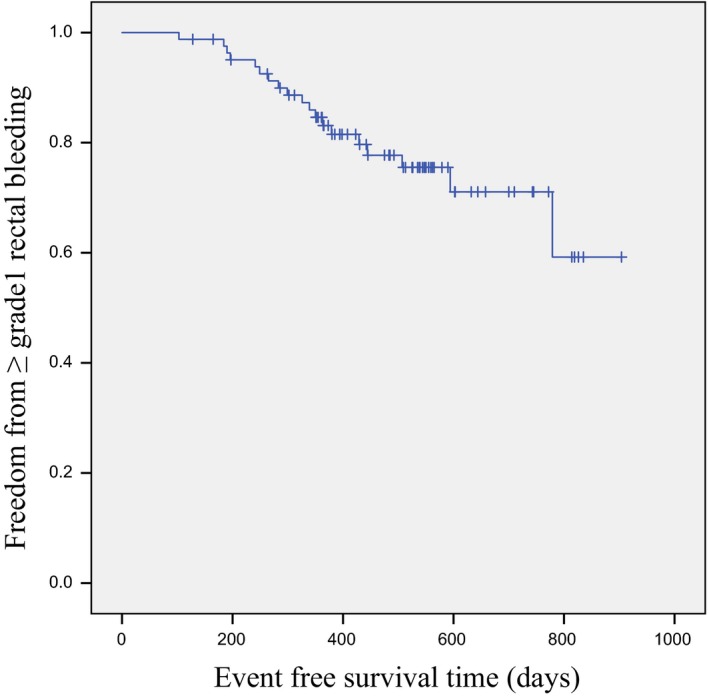
Kaplan‐Meier plot for ≥Grade 1 rectal bleeding.

Grade ≥1 rectal bleeding was observed in 19 (23.2%) patients. Event‐free survival (EFS) for Grade ≥1 late rectal bleeding was calculated from the last day of radiotherapy. Patients who were free from events were censored at the date of last follow‐up. As shown in Figure 1, Kaplan–Meier curves for EFS regarding Grade ≥1 late rectal bleeding were generated. In univariate analysis, improved EFS for Grade ≥1 late rectal bleeding was significantly associated with a rectal volume <35.33 cc, a mean rectal dose <45.72 Gy and a rectal V30 < 2.91%, V40 < 55.45%, V50 < 39.54%, V60 < 25.30% and V70 < 9.07% (Table 3). Multivariate analysis was performed for those factors which had a significant association when evaluated using univariate analysis. There was strong collinearity among the dosimetric factors mentioned above with the exception of rectal volume. Therefore, they could not be tested simultaneously and were tested independently with rectal volume. On multivariate analysis, all of the above factors remained significant with the exception of a rectal V70 < 9.07% (Table 4).

**Table 3:** Univariate analysis for ≥Grade 1 late rectal bleeding

	Patients, n	1‐year EFS (%)	*P*
Age (years)			
<64	8	100	0.159
≥64	74	82.9	
T stage			
≤T2b	35	81.6	0.866
≥T2c	47	84.1	
D'Amico classification			
Low–intermediate	24	78.9	0.637
High	58	85.2	
Hormonal therapy			
Yes	78	82.2	0.977
No	4	100	
Hypertension			
Yes	33	80.1	0.880
No	49	85.0	
Diabetes			
Yes	12	74.1	0.290
No	70	84.8	
Use of anticoagulants or antiaggregants			
Yes	18	76.5	0.459
No	64	85.0	
Pelvic nodal irradiation			
Yes	42	81.6	0.383
No	40	84.5	
Prostate volume (cc)			
<23.32	45	85.6	0.447
≥23.32	37	80.6	
PTV volume (cc)			
<76.08	7	85.7	0.921
≥76.08	75	83.0	
Rectal volume (cc)			
<35.33	60	87.6	0.053
≥35.33	22	70.9	
Rectal maximal dose (Gy)			
<77.07	30	86.2	0.346
≥77.07	52	81.2	
Rectal mean dose (Gy)			
<45.72	33	96.0	0.002
≥45.72	49	75.1	
Rectal V30 (%)			
<72.91	28	95.5	0.006
≥72.91	54	77.0	
Rectal V40 (%)			
<55.45	32	96.2	0.006
≥55.45	50	74.7	
Rectal V50 (%)			
<39.54	28	95.5	0.005
≥39.54	54	76.7	
Rectal V60 (%)			
<25.30	23	94.7	0.019
≥25.30	59	78.7	
Rectal V70 (%)			
<9.07	16	92.9	0.086
≥9.07	66	80.7	
Rectal V75 (%)			
<1.47	21	83.9	0.329
≥1.47	61	83.0	
Rectal V76 (%)			
<0.36	18	88.2	0.218
≥0.36	64	81.6	

EFS, event‐free survival of patients with ≥Grade 1 late rectal bleeding; PTV, planning target volume; V30–V76, volume receiving ≥30–76 Gy.

**Table 4:** Multivariate analysis for ≥Grade 1 late rectal bleeding

Characteristics	HR	95% CI	*P*
Analysis 1			
Rectal mean dose (Gy)	13.303	1.769–100.068	0.012
Rectal volume (cc)	2.697	1.081–6.727	0.033
Analysis 2			
Rectal V30 (%)	10.038	1.337–75.373	0.025
Rectal volume (cc)	2.463	0.987–6.148	0.053
Analysis 3			
Rectal V40 (%)	6.790	1.554–29.662	0.011
Rectal volume (cc)	2.839	1.136–7.092	0.026
Analysis 4			
Rectal V50 (%)	11.665	1.546–88.007	0.017
Rectal volume (cc)	2.905	1.163–7.258	0.022
Analysis 5			
Rectal V60 (%)	8.680	1.152–65.426	0.036
Rectal volume (cc)	2.760	1.103–6.909	0.030
Analysis 6			
Rectal V70 (%)	6.105	0.802–46.456	0.081
Rectal volume (cc)	2.863	1.138–7.199	0.025

HR, hazard ratio; CI, confidence interval; PTV, planning target volume; V30–V76, volume receiving ≥ 30–76 Gy.

### Patients with Grade 2–3 late rectal bleeding

Grade 2–3 rectal bleeding was observed in five (6.1%) patients. Univariate analysis indicated that rectal bleeding was significantly associated with use of anticoagulants or antiaggregates, and rectal V30 and V40 (Table 5).

**Table 5:** Factors associated with Grade 2–3 late rectal bleeding

	Mean (range)		*P*
	Grade 0–1 (*n* = 77)	Grade 2–3 (*n* = 5)	
Clinical factor			
Age (years)	72 (57–85)	75 (70–78)	0.406
T stage (≤T2b/≥T2c)	32/45	3/2	0.646
D'Amico classification (≤Intermediate/high)	21/56	3/2	0.147
Hormonal therapy (yes/no)	73/4	5/0	1.000
Hypertension (yes/no)	30/47	3/2	0.387
Diabetes (yes/no)	11/66	1/4	0.556
Use of anticoagulants or antiaggregants (yes/no)	14/63	4/1	0.007
Pelvic nodal irradiation (yes/no)	38/39	2/3	1.000
Dosimetric factor			
Prostate volume (cc)	25.87 (10.63–74.90)	20.75 (14.92–25.99)	0.380
PTV volume (cc)	104.19 (56.75–203.01)	93.02 (77.96–105.49)	0.373
Rectal volume (cc)	30.15 (14.36–63.41)	29.98 (21.51–38.44)	0.963
Rectal maximal dose (Gy)	77.23 (73.99–78.98)	70.61 (76.86–78.46)	0.297
Rectal mean dose (Gy)	46.75 (29.70–62.08)	51.41 (48.71–55.31)	0.075
Rectal V30 (%)	77.90 (38.45–99.94)	92.54 (84.72–99.21)	0.021
Rectal V40 (%)	58.76 (28.43–96.03)	71.86 (62.21–83.37)	0.041
Rectal V50 (%)	43.46 (20.12–86.69)	50.45 (41.98–61.83)	0.208
Rectal V60 (%)	29.40 (12.20–60.30)	33.57 (25.46–40.53)	0.323
Rectal V70 (%)	13.83 (0.98–29.70)	16.65 (10.57–23.56)	0.272
Rectal V75 (%)	3.78 (0–12.61)	5.43 (2.49–11.56)	0.214
Rectal V76 (%)	1.64 (0–7.87)	2.48 (0.67–6.54)	0.309

PTV, planning target volume; V30–V76, volume receiving ≥30–76 Gy.

## Discussion

We investigated factors associated with late rectal bleeding following IMRT using TomoTherapy for prostate cancer. Our results suggested that the volume, mean dose and rectal V30, V40, V50 and V60 were significant predictors for Grade ≥1 late rectal bleeding. As for Grade 2–3 rectal bleeding, the use of anticoagulants or antiaggregates, and the rectal V30 and V40 were predictive factors. Rectal dose‐volumes >V70, which represented the volume of the highest dose, were not predictive of any grade of late rectal bleeding in this study.

In line with our study, Jackson et al.^12^ have also reported the importance of irradiated rectal volume regarding the intermediate dose in CRT. They found that the volume of the anatomic rectal wall exposed to intermediate doses (40–50 Gy) as well as to ˃70 Gy were independently correlated with Grade ≥2 bleeding. However, the importance of the intermediate dose concerning rectal bleeding is not considered in the mainstay of dose‐escalated conformal treatment, such as 3D‐CRT and IMRT. Several investigators have demonstrated the serial‐like behaviour of rectal bleeding, especially when considering severe bleeding; this means that relatively small fractions of rectum irradiated at ‘high’ doses are the main cause of bleeding.^7^, ^8^, ^9^, ^10^ Söhn et al.^7^ found that the volume receiving >73.7 Gy showed the most significant correlation with bleeding. Fellin et al.^8^ investigated moderate/severe late rectal toxicity (Grade 2–3). They reported that the risk of rectal bleeding was reduced by limiting the V75 Gy to <5%. The finding of Fiorino et al. and Peeters et al. were also in accordance with these reports.^9^, ^10^ All of these authors stated that the rectum acts as a ‘serial’ organ when considering late bleeding.

We suggest there are two main reasons why we could not find serial‐like behaviour regarding late rectal bleeding. One reason was that we aimed at adhering to rectal dose constraints as strictly as possible, and particularly prioritised high‐dose constraints such as the rectal V75 or V76. However, less attention might have been paid to intermediate dose constraints relative to high‐dose constraints. The other reason was that we considered not only moderate to severe rectal bleeding (Grade 2–3) but also mild rectal bleeding (Grade 1). In contrast, those studies mentioned above considered only moderate to severe rectal bleeding (Grade 2–3). These differences make the findings of our study distinct from the other studies.

Our finding concerning the prognostic significance of intermediate doses brings a new understanding of its importance in decreasing any grade of rectal bleeding. While maintaining strict attention on high doses is obviously essential, doing so with intermediate doses is also important.

Recently, based on our experience, we developed several strategies to achieve both dose reduction in the rectum and conformal dose distribution around the target. We now use some virtual structures in the anterior part of the rectum, in which the dose is more strictly restricted relative to other regions of the rectum. These virtual structures are effective in regulating low to high level doses to the whole rectum. In addition, the TomoTherapy system uses daily MVCT imaging guidance, which enables precise set up based on patient anatomy, even if the PTV margin is restricted. Therefore, we have adapted reduced PTV margins (0.8 cm with the exception of the posterior margin which was 0.5 cm) in patients with T1 or T2 disease, whose risk of peri‐prostatic extension was limited, to suppress the dose to rectum and bladder. We assumed that these strategies might reduce the incidence of rectal bleeding.

A variety of intensity modulated delivery methods have been used in conjunction with image‐guided radiotherapy. TomoTherapy is a unique form of IMRT that may offer improvement in the treatment plan. Shah et al.^13^ investigated the treatment advantage of TomoTherapy over static field IMRT. They found that TomoTherapy achieved reduced dose exposure in the bladder and rectum and an improvement in target homogeneity. Wolff et al.^14^ compared volumetric arc therapy (VMAT) with TomoTherapy, step‐and‐shoot IMRT and 3D‐CRT for prostate cancer. All intensity modulated techniques significantly improved treatment quality when compared with 3D‐CRT. They found that TomoTherapy provided the best rectal dose sparing. However, VMAT was superior in relation to treatment efficiency because TomoTherapy requires a larger monitor unit (MU), which results in longer treatment times.^14^ Tsai et al.^15^ also investigated the planning advantages among these intensity modulated strategies; they found that TomoTherapy achieved better dose conformity of the target and rectal sparing, but required the highest MU and the longest delivery time. Although, there are pros and cons concerning each treatment modality, longer follow‐up is needed to assess treatment outcomes in these intensity modulated strategies.

We found that the use of anticoagulants or antiaggregates had prognostic value regarding moderate to severe late rectal bleeding. It seems reasonable to conclude that the use of these medications can influence the risk of bleeding. In agreement with our study, Choe et al.^16^ reported that the use of anticoagulation therapy was associated with greater rates of Grade 3 or worse bleeding toxicity. Given this risk, special attention is warranted when considering rectal dose constraints in patients receiving these medications. Several clinical and dosimetric factors that can possibly predict rectal bleeding have been studied; these factors are not always associated with this adverse effect. According to the most recent studies, genetic factors are becoming an issue. A genome‐wide association study has identified novel genetic markers for rectal bleeding following radiotherapy; these markers could be used to better tailor therapy for individual patients.^17^, ^18^

Finally, we recognise that our study has limitations such as small number of patients and limited follow‐up time. Rectal bleeding secondary to radiotherapy could occur even after 2 years from completion of radiotherapy.^19^ Long follow‐up is needed for these patients.

## Conclusions

Our results suggested that the volume of intermediate doses to the rectum was a significant predictor of mild to severe late rectal bleeding (Grade ≥1). Rectal dose‐volumes >V70, which represented the volume involving the highest doses, were not predictive in this study. Maintaining strict attention on high as well as intermediate doses to the rectum is important in reducing the incidence of any grade of rectal bleeding. These findings should be confirmed in a more extensive prospective study.

## Conflict of interest

The authors declare no conflict of interest.

## References

[1] Kuban DA , Tucker SL , Dong L , et al. Long‐term results of the M. D. Anderson randomized dose‐escalation trial for prostate cancer. Int J Radiat Oncol Biol Phys 2008; 70: 67–74. 17765406 10.1016/j.ijrobp.2007.06.054

[2] Zietman AL , DeSilvio ML , Slater JD , et al. Comparison of conventional‐dose vs high‐dose conformal radiation therapy in clinically localized adenocarcinoma of the prostate: A randomized controlled trial. JAMA 2005; 294: 1233–9. 16160131 10.1001/jama.294.10.1233

[3] Zelefsky MJ , Yamada Y , Fuks Z , et al. Long‐term results of conformal radiotherapy for prostate cancer: Impact of dose escalation on biochemical tumor control and distant metastases‐free survival outcomes. Int J Radiat Oncol Biol Phys 2008; 71: 1028–33. 18280056 10.1016/j.ijrobp.2007.11.066

[4] Cahlon O , Hunt M , Zelefsky MJ . Intensity‐modulated radiation therapy: Supportive data for prostate cancer. Semin Radiat Oncol 2008; 18: 48–57. 18082588 10.1016/j.semradonc.2007.09.007

[5] Zelefsky MJ , Fuks Z , Happersett L , et al. Clinical experience with intensity modulated radiation therapy (IMRT) in prostate cancer. Radiother Oncol 2000; 55: 241–9. 10869739 10.1016/s0167-8140(99)00100-0

[6] Fiorino C , Valdagni R , Rancati T , Sanguineti G . Dose–volume effects for normal tissues in external radiotherapy: Pelvis. Radiother Oncol 2009; 93: 153–67. 19765845 10.1016/j.radonc.2009.08.004

[7] Söhn M , Yan D , Liang J , Meldolesi E , Vargas C , Alber M . Incidence of late rectal bleeding in high‐dose conformal radiotherapy of prostate cancer using equivalent uniform dose‐based and dose‐volume‐based normal tissue complication probability models. Int J Radiat Oncol Biol Phys 2007; 67: 1066–73. 17258870 10.1016/j.ijrobp.2006.10.014 PMC1991336

[8] Fellin G , Fiorino C , Rancati T , et al. Clinical and dosimetric predictors of late rectal toxicity after conformal radiation for localized prostate cancer: Results of a large multicenter observational study. Radiother Oncol 2009; 93: 197–202. 19828205 10.1016/j.radonc.2009.09.004

[9] Fiorino C , Fellin G , Rancati T , et al. Clinical and dosimetric predictors of late rectal syndrome after 3D‐CRT for localized prostate cancer: Preliminary results of a multicenter prospective study. Int J Radiat Oncol Biol Phys 2008; 70: 1130–7. 17881142 10.1016/j.ijrobp.2007.07.2354

[10] Peeters ST , Hoogeman MS , Heemsbergen WD , Hart AA , Koper PC , Lebesque JV . Rectal bleeding, fecal incontinence, and high stool frequency after conformal radiotherapy for prostate cancer: Normal tissue complication probability modeling. Int J Radiat Oncol Biol Phys 2006; 66: 11–19. 16757129 10.1016/j.ijrobp.2006.03.034

[11] RTOG Contouring Atlas, Prostate Pelvic Lymph Nodes, Pelvic Lymph Node Volumes for Prostate Cancer Atlas. Available from: https://www.rtog.org/CoreLab/ContouringAtlases/ProstatePelvicLymphNodes.aspx (Accessed 9 March 2011).

[12] Jackson A , Skwarchuk MW , Zelefsky MJ , et al. Late rectal bleeding after conformal radiotherapy of prostate cancer. II. Volume effects and dose‐volume histograms. Int J Radiat Oncol Biol Phys 2001; 49: 685–98. 11172950 10.1016/s0360-3016(00)01414-0

[13] Shah AP , Chen SS , Strauss JB , et al. A dosimetric analysis comparing treatment of low‐risk prostate cancer with TomoTherapy versus static field intensity modulated radiation therapy. Am J Clin Oncol 2009; 32: 460–6. 19564784 10.1097/COC.0b013e3181967d89

[14] Wolff D , Stieler F , Welzel G , et al. Volumetric modulated arc therapy (VMAT) vs. serial tomotherapy, step‐and‐shoot IMRT and 3D‐conformal RT for treatment of prostate cancer. Radiother Oncol 2009; 93: 226–33. 19765846 10.1016/j.radonc.2009.08.011

[15] Tsai CL , Wu JK , Chao HL , Tsai YC , Cheng JC . Treatment and dosimetric advantages between VMAT, IMRT, and helical tomotherapy in prostate cancer. Med Dosim 2011; 36: 264–71. 20634054 10.1016/j.meddos.2010.05.001

[16] Choe KS , Jani AB , Liauw SL . External beam radiotherapy for prostate cancer patients on anticoagulation therapy: How significant is the bleeding toxicity? Int J Radiat Oncol Biol Phys 2010; 76: 755–60. 19464123 10.1016/j.ijrobp.2009.02.026

[17] Kerns SL , Stock RG , Stone NN , et al. Genome‐wide association study identifies a region on chromosome 11q14.3 associated with late rectal bleeding following radiation therapy for prostate cancer. Radiother Oncol 2013; 107: 372–6. 23719583 10.1016/j.radonc.2013.05.001 PMC3787843

[18] Fachal L , Gómez‐Caamaño A , Peleteiro P , et al. Association of a XRCC3 polymorphism and rectum mean dose with the risk of acute radio‐induced gastrointestinal toxicity in prostate cancer patients. Radiother Oncol 2012; 105: 321–8. 23075580 10.1016/j.radonc.2012.09.013

[19] Cahlon O , Zelefsky MJ , Shippy A , et al. Ultra‐high dose (86.4 Gy) IMRT for localized prostate cancer: Toxicity and biochemical outcomes. Int J Radiat Oncol Biol Phys 2008; 71: 330–7. 18164858 10.1016/j.ijrobp.2007.10.004

